# Immunity in SARS-CoV-2 Infection: Clarity or Mystery? A Broader Perspective in the Third Year of a Worldwide Pandemic

**DOI:** 10.1007/s00005-023-00673-0

**Published:** 2023-02-21

**Authors:** Katarzyna Kapten, Krzysztof Orczyk, Elzbieta Smolewska

**Affiliations:** 1 Department of Pediatric Cardiology and Rheumatology, Central Teaching Hospital of Medical University of Lodz, Lodz, Poland; 2 Department of Pediatric Cardiology and Rheumatology, Medical University of Lodz, Sporna 36/50, 91-738 Lodz, Poland

**Keywords:** SARS-CoV-2, Innate immunity, Humoral response, Cellular response, Vaccinations, Immunosuppression

## Abstract

Severe acute respiratory syndrome coronavirus-2 (SARS-CoV-2) and its mechanisms have been thoroughly studied by researchers all over the world with the hope of finding answers that may aid the discovery of new treatment options or effective means of prevention. Still, over 2 years into the pandemic that is an immense burden on health care and economic systems, there seem to be more questions than answers. The character and multitude of immune responses elicited in coronavirus disease 2019 (COVID-19) vary from uncontrollable activation of the inflammatory system, causing extensive tissue damage and consequently leading to severe or even fatal disease, to mild or asymptomatic infections in the majority of patients, resulting in the unpredictability of the current pandemic. The aim of the study was to systematize the available data regarding the immune response to SARS-CoV-2, to provide some clarification among the abundance of the knowledge available. The review contains concise and current information on the most significant immune reactions to COVID-19, including components of both innate and adaptive immunity, with an additional focus on utilizing humoral and cellular responses as effective diagnostic tools. Moreover, the authors discussed the present state of knowledge on SARS-CoV-2 vaccines and their efficacy in cases of immunodeficiency.

## Introduction

Since the end of 2019, when the novel coronavirus emerged in Wuhan, China, there has been an unprecedented incentive of researchers, doctors, and scientists from all over the world attempting to get the full picture of the severe acute respiratory syndrome coronavirus-2 (SARS-CoV-2) infection. Soon it became clear that only a complete and comprehensive understanding of the new virus could provide the healthcare systems and governments with the means to not only limit the spread of the disease but also provide necessary data for drugs and vaccine development. Even though, during the course of the pandemic, these aims proved to be more challenging than initially suspected, with the complexity of the coronavirus disease 2019 (COVID-19) and its various clinical presentations (Dong et al. 2020). Nearly three years into the pandemic we ended up with more detailed knowledge of SARS-CoV-2 than possibly any other virus throughout history. As of today, the current pandemic is the third serious epidemic caused by beta-coronavirus since 2002 preceded by severe acute respiratory syndrome (SARS) and Middle East respiratory syndrome (MERS) (Gusev et al. 2022). Since these pathogens bear high resemblance in their capability of infecting multiple cell types in several organ systems (Gu et al. 2005), the discoveries made regarding SARS-CoV-2 may not only benefit us in the current epidemiological situation but also in the years to come, as new challenges may arise for medical professionals.

In this narrative review, the authors attempt to systematize the data on both innate and adaptive immunity to the SARS-CoV-2 infection (Table 1). The scope of this paper is to cover immune mechanisms, from the most indispensable in the first line of defense against pathogens, like the role of combined influx of cytokines, macrophages and interferons (IFNs), which significance was well noted by Silva et al. (2022), to a more comprehensive insight into both humoral and cellular immunity. As stated by Vályi-Nagy et al. (2022) in a review on adaptive immunity in SARS-CoV-2, only a coordinated and balanced work of both immune systems guarantee overcoming the infection. Additionally, the article explores the immune reactions to the newly developed and widely used vaccines, pointing out the limitations of the sole assessment of antibodies titers and significance of cellular immunity as was signaled in similar reviews concentrating solely on vaccinations to SARS-CoV-2 (Laidlaw and Ellebedy 2022; Sadarangani et al. 2021). The authors aimed to add to the current literature a comprehensive overview of a current state of knowledge regarding SARS-CoV-2 virus with its implications on available testing methods and vaccination efficacy, with an additional focus on immunodeficient patients.

**Table 1:** Main immunity mechanism in SARS-CoV-2 infection mentioned in the paper

Main immunity mechanisms in SARS-CoV-2 infection		
	Innate immunity	Adaptive immunity
Humoral components	Complement	B-cell produced antibodies mediated immunity
	Coagulation-fibrinolysis cascades	
	Proteins	
	Cytokines: chemokines, ILs, IFNs, TNF	
	Naturally occurring antibodies	
	Macrophages and monocytes	
Cellular components	NK cells	T cells mediated immunity (mainly CD8^+^)
	Nonspecific leucocytes	IL (IL-6, IL-17) produced by T cells

*IL* interleukin, *IFN* interferons, *TNF* tumor necrosis factor, *NK* natural killer

## General Characteristics of SARS-CoV-2

**Fig. 1:**
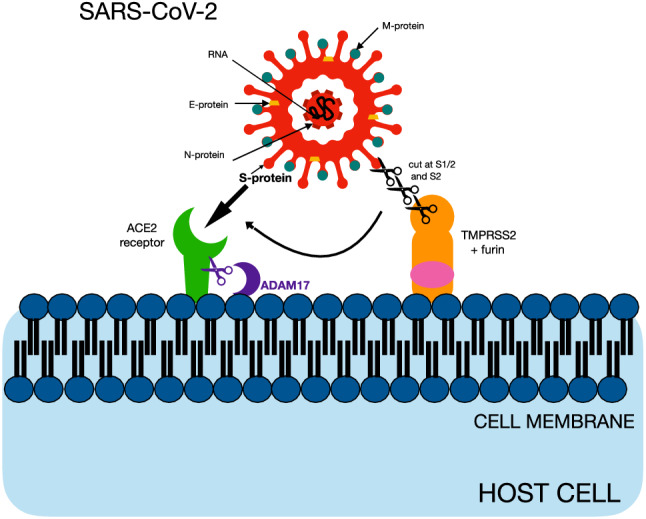
Structure and cell entry mechanism of SARS-CoV-2. The figure illustrates the architecture of severe acute respiratory syndrome coronavirus-2 (SARS-CoV-2). It is characterized by the presence of four structural proteins: S (spike), E (envelope), M (membrane), and N (nucleocapsid), which interacts with the genomic RNA. Virus communication with the host cell is mediated by S protein and facilitated mainly by angiotensin-converting enzyme 2 (ACE2) receptors, which are highly expressed on the cell membranes of many tissues and organs. The binding of the S glycoprotein to the ACE2 receptor is followed by the proteolytic cut at the S1/S2 site of the S glycoprotein by the host protease furin and cleavage at the S2 site by transmembrane serine protease 2 (TMPRSS2), subsequently. ACE2 presence on the plasma membranes is regulated by the shedding of the protein, promoted by A disintegrin and metalloprotease 17 (ADAM17)

SARS-CoV-2 is an enveloped, positive-sense single-stranded RNA virus. It shares main structural and molecular characteristics with other coronaviruses, such as the presence of four structural proteins: S (spike), E (envelope), M (membrane), and N (nucleocapsid) that are critical for binding with cellular receptors, viral replication, and pathogenicity (Huang and Wang 2021). The M glycoprotein is responsible for the formation and stability of the viral envelope and the N protein interacts with the genomic RNA. Communication of the virus with the host cell is facilitated by angiotensin converting enzyme 2 (ACE2) receptors and is mediated by the S protein of the virus. ACE2 receptors are highly expressed on the cell surface of many tissues and organs, mainly the respiratory tract mucosa, but also the myocardial surface and digestive system mucosa (Lei et al. 2021; Rizzo et al. 2020). The S1 region of the S protein is responsible for binding to the host cell receptor, while the S2 region is responsible for the fusion of the viral particles and genome with the host cell (Gadanec et al. 2021). Binding of the viral S glycoprotein to the ACE2 receptor on the cell's surface must be followed by the proteolytic cut at the S1/S2 site of the S glycoprotein by the host protease furin. S protein has to be cleaved by the host factor, transmembrane serine protease 2 (TMPRSS2) at the S2 site in order to expose a fusion peptide which is an essential step for viral fusion with the host cell. These processes occur sequentially, with the cleavage at the S1/S2 site occurring first followed by the subsequent cleavage at S2′. TMPRSS2 and furin are both essential for proteolytic activation of SARS-CoV-2 (Bestle et al. 2020). ACE2 presence on the plasma membranes is regulated by A disintegrin and metalloprotease 17 (ADAM17) which promotes the shedding of the protein (Rizzo et al. 2020) (Fig. 1). However, as the newest research shows, ACE2 receptors are not imperative for SARS-CoV-2 infection. Not only does the presence of specific co-receptors enables the virus to infect cells with low ACE2 expression on membranes, but there is also growing evidence of the existence of alternative ACE2 pathways for target cell infection, utilizing immune receptors like neuropilin-1, C-lectin type receptors, Toll-like receptors and the non-immune receptor glucose regulated protein 78 (Amraei et al. 2021; Choudhury and Mukherjee 2020; Gadanec et al. 2021; Gao et al. 2020; Ibrahim et al. 2020). The variety of pathways in which SARS-CoV-2 infects human tissues may explain its high affinity and robust spread through the population.

As a result of replication errors mediated by RNA polymerase and reverse transcriptase enzymes, SARS-CoV-2 as an RNA virus has a substantially higher mutation rate than DNA viruses. Thus, the continuous transmission and the high rate of replication errors of the virus have led to the emergence of many mutations across geographical regions, mainly observed in the receptor-binding domain in the S-glycoprotein. Therefore, the identification of all SARS-CoV-2 variants and specification of their pathophysiology is merely impossible (Gusev et al. 2022; Huang and Wang 2021; Santacroce et al. 2021).

## Innate Immunity

As an effective adaptive immune response to SARS-CoV-2 could not be expected to occur until at least 2–3 weeks after the initial exposure, very efficient control of the infection was observed in the vast majority of first-time infected population which shows that the added value of the innate immune response cannot be overlooked. Antiviral innate immunity reaction includes both humoral components, such as complement, coagulation-fibrinolysis cascades, proteins, chemokines, and naturally occurring antibodies, as well as cellular components like natural killer (NK) cells and other nonspecific phagocytic and cytolytic leukocytes (Boechat et al. 2021; Weber 2021).

Interleukin (IL)-6 was identified as one of the first potentially pathogenic factors in the development of acute respiratory distress syndrome (ARDS) in the course of COVID-19. Being a part of both innate and adaptive immunity, IL-6 has a crucial role in the initial response to pathogens and ischemic injury by producing acute phase proteins. Additionally, it directs immune cell differentiation and takes part in immunoglobulin production, by having stimulatory effects on both B cells and T cells, thus, promoting chronic inflammation (Gabay 2006). The uncontrolled production of IL‐6 is a common characteristic of autoimmune and autoinflammatory diseases. The introduction of anti-IL-6 receptor monoclonal antibodies has resulted in great therapeutical success in rheumatic diseases (Jordan et al. 2020; Jordan 2021). The excessive IL-6 production in SARS-CoV-2 infection is reported to cause a cytokine storm, leading to endothelial cell damage, capillary leak, and eventually ARDS (Jordan 2021). Additionally, it was proved to be a risk factor for the requirement of mechanical ventilation in COVID-19 patients (Herold et al. 2020). For this reason, preventing excessive IL‐6 production or targeting IL-6 receptors, was considered a viable treatment approach that could potentially limit morbidity and mortality in the context of SARS-CoV-2 infection. However, while initial results from observational studies were very promising (Guaraldi et al. 2020; Price et al. 2020; Somers et al. 2021; Wise 2020), they were not always followed by comparable results in clinical trials (García-Lledó et al. 2022; Stone et al. 2020).

Furthermore, patients with a severe course of SARS-CoV-2 infection were found to have significantly elevated serum levels of not only IL-6 but also several other pro-inflammatory cytokines, including IL-1β, IL-2, IL-8, IL-17, as well as granulocyte and granulocyte–macrophage colony-stimulating factors, IFN-γ-induced protein 10, monocyte chemoattractant protein-1 and tumor necrosis factor. The newest studies stress the role of IL-17, which high levels in both nasal swabs and lung autopsies of patients with fatal SARS-CoV-2 infection were associated with higher levels of proinflammatory cytokines. Hence, creating a positive feedback loop intensifies the impact of IL-17 and causes a possibly self-sustaining process of IL-17 secretion (Sharif-Askari et al. 2022). Furthermore, it can lead to a cytokine storm, which, through a further positive feedback circuit can cause multiple organ failure with extensive tissue damage to the heart, liver, and kidneys, as well as substantial pulmonary pathology with neutrophils and macrophages infiltration, leading to diffused alveolar damage. Autopsies done on patients who died from COVID-19 revealed a high infiltration of macrophages within the areas of bronchopneumonia (Barton et al. 2020). In addition, laboratory parameters found in patients with severe SARS-CoV-2 infection, such as abnormally high levels of C-reactive protein, d-dimers, and ferritin, together with coagulopathy and hypoproteinemia are characteristic features of hyperinflammation known under the umbrella term of macrophage activation syndrome (MAS) or secondary hemophagocytic lymphohistiocytosis (HLH) (Cao 2020; McGonagle et al. 2020). It is a life-threatening condition characterized by pancytopenia, liver failure, hyperferritinemia, coagulopathy, and neurologic symptoms due to uncontrolled proliferation of well-differentiated macrophages, leading to cytokine overproduction and hemophagocytosis. Macrophages which are, essentially, the tissue analogues of monocytic cells, are classified according to their activation pathways. While both classically (M1), and alternatively polarized macrophages (M2) can suppress SARS-CoV-2 infection, M1 activated mainly by IFN-γ or lipopolysaccharides, and non-activated macrophages (M0) have been found to overstimulate the inflammatory response and lead to lung cells apoptosis. Adversely, M2 activated by IL-3 or IL-13, can be generally characterized as anti-inflammatory agents (Lian et al. 2022; Świdrowska-Jaros et al. 2016). Secondary HLH has been already observed during lethal influenza pandemics and previous SARS and MERS coronavirus outbreaks (Gómez-Rial et al. 2020). The MAS-like disease which may develop in the course of SARS-CoV-2 infection is mainly limited to the lungs and characterized by extensive pulmonary microthrombosis rather than disseminated intravascular coagulation that typically follows, making it difficult to discern from ARDS (McGonagle et al. 2020).

As we already know, monocytes and macrophages fuel the cytokine storm observed in COVID-19 patients, therefore they are one of the key elements leading to ARDS and subsequently poor prognosis (Schiuma et al. 2022). The newest research on the topic indicates, that the replication of SARS-CoV-2 in human lung macrophages activates inflammasomes which initiate an inflammatory cascade, eventually resulting in pyroptosis of macrophages and contributing to the downstream type-I-IFN response. While the inflammasome activation stops the virus replication, the excessive inflammation that occurs through this mechanism alongside the dysregulated IFN response may lead to an over-exuberant inflammatory reaction that we observe in COVID-19 (Sefik et al. 2022).

Neutrophils may as well play an important role in the inflammatory response to SARS-CoV-2, by promoting organ injury and coagulopathy (immunothrombosis) via direct tissue infiltration and formation of neutrophil extracellular traps (NETs) in a process known as NETosis (Middleton et al. 2020; Zuo et al. 2020). Activated through inflammasome pathways, CD14^+^ monocytes accomplish phagocytosis of dead neutrophils and promote NETosis in the lungs, leading to decreased lymphocyte/neutrophils ratio and therefore, as ample pieces of evidence suggest, a higher risk of death (Roy et al. 2021).

Lymphopenia was found to be one of the hallmarks of SARS-CoV-2 infection, with lower lymphocyte counts, including three main populations, T, B, and NK cells, closely linked to bad prognosis (Antonioli et al. 2020; Moss 2022; Tan et al. 2020; Wang et al. 2020). NK cells are yet another component of the dysregulated immune system that proved to play a pivotal role in the pathogenesis of COVID-19. The evidence shows that SARS-CoV-2 infection might compromise the innate antiviral immunity by exhaustion of NK cells functions (Antonioli et al. 2020; Market et al. 2020). Simultaneously, an increase in NK cells count and a decrease in NK cell receptor (NKG2A) expression were observed (Bortolotti et al. 2020; Market et al. 2020; Yaqinuddin and Kashir 2020). Interestingly, a notable reduction of NK cells activation and their ability to degranulate was observed while no direct effect of the viral proteins on NK cells activation was proved in vivo if the same process was evaluated in lung epithelial cells (Bortolotti et al. 2020). It has also been postulated that SARS-CoV-2 infection can compromise innate immunity even after the patient’s recovery.

A delayed and inadequate IFN response to COVID-19 contributed further to the unrestrained viral replication and therefore tissue damage. Upon SARS-CoV-2 infection, signaling cascades are activated, which results in IFN production by epithelial and endothelial cells, alveolar macrophages, NK cells, dendritic cells, and inflammatory monocyte-macrophages. SARS-CoV-2 developed mechanisms to weaken the IFN response, such as proteins that interspersed between the structural genes of the virus, antagonizing or evading the IFN response, contributing to its delayed expression. As IFNs are a wide group of cytokines that are divided into three main groups IFN-I, IFN-II, and IFN-III, a multitude of research was performed attempting to correlate the release of specific IFNs to the severity of SARS-CoV-2 infection. However, with contradicting data on that matter, an interpatient variability in IFN response should be assumed. A severe course of the disease was found to be accompanied by both prolonged or insufficient IFN-I and IFN-3 production depending on the individual, while other data suggests an upregulation of IFN-II production in critically ill patients (Galani et al. 2021; Hadjadj et al. 2020; Huang et al. 2020; Lowery et al. 2021; Lucas et al. 2020b). What remains evident is that early IFN response can be protective in the acute phase of the infection while a disrupted IFN production is a risk factor for severe COVID-19 (Bastard et al. 2020; Lowery et al. 2021).

## Adaptive Immunity

### Humoral Immunity

While the humoral response to SARS-CoV-2 has been one of the most thoroughly investigated components of the immunity against the virus, there is still some controversy over its role in the defense against subsequent COVID-19, as well as its place in the assessment of both post-vaccination and post-exposure immunity.

The seroconversion of IgM and IgA antibodies can vary between 4 and 6 or even 3–12 days after the onset of the disease, for IgG antibodies between 5 and 18 days, depending on the source and individual. With a substantially high positive detection rate of SARS-CoV-2 utilizing IgM antibody ELISA assays and the possibility of achieving even higher sensitivity by combining antibodies detection with polymerase chain reaction, IgM antibodies testing has become a viable accessory in the diagnosis of COVID-19 in its acute phase (Choteau et al. 2022; Guo et al. 2020; Zhao et al. 2020).

Numerous research show early appearance and higher titers of anti-SARS-CoV-2 antibodies in patients suffering from severe forms of the disease compared with milder cases, suggesting not only the relation between the magnitude of IgG response to both viral load and disease severity but also raising the possibility of a pathological role of antibody response (Boechat et al. 2021; Choteau et al. 2022; Lynch et al. 2021; Zhang et al. 2020; Zhao et al. 2020). As has been observed in other infections, there is a potential antibody-dependent enhancement mechanism of SARS-CoV-2, which is characterized as antibody-mediated augmentation of viral entry and initiation of a severe inflammatory response (Arvin et al. 2020; Cao 2020; Lee and Oh 2021). However, Lucas et al. (2021) found that deceased patients did not have overall higher titers of antibodies than recovered individuals, suggesting that in severe cases, there is a robust, but short-lived immune response. Therefore, slow kinetics and delayed production of neutralizing antibody may be the key to the impaired viral control in fatal cases of SARS-CoV-2 (Lucas et al. 2020a).

While studies on COVID-19 patients suffering from primary humoral immunodeficiencies (such as common variable immunodeficiency) confirm that B-cell response plays an important role in the course of the infection (Quinti et al. 2020), a full recovery in patients with immune deficits have been observed. Cases of patients with agammaglobulinemia show that even with a higher risk of developing pneumonia in the course of SARS-CoV-2 (Soresina et al. 2020), mild symptoms and eventually favorable outcome of the disease was attained (Quinti et al. 2020). Similarly, the report on patients with common variable immunodeficiencies suggested that they are at a standard risk for developing severe disease (Cohen et al. 2021). Moreover, studies suggest that in cases of limited humoral responses, the role of T-cell immunity should not be overlooked (Bange et al. 2021).

A further concern is the longevity of humoral immunity and its critical role in protection from pathogen re-infection, since the existence of controversial data concerning the persistence of antibodies titers after SARS-CoV-2 exposure (Isho et al. 2020). While some studies have shown long-lasting and stable levels of neutralizing antibodies (Al-Naamani et al. 2021; Choteau et al. 2022) others have described a rapid decline of anti-SARS-CoV-2 IgG titers in a few months after the disease has resolved. A prompt drop in antibody levels is mostly associated with mild or asymptomatic disease, which cannot be ignored since they account for the majority of COVID-19 cases (Ibarrondo et al. 2020; Long et al. 2020).

While most of the standard serological testing in SARS-CoV-2 focuses on IgM and IgG antibodies, IgA response to coronavirus infection was found to be stronger and more persistent than IgM does (Padoan et al. 2020). According to Sterlin et al. (2021), IgA contributed to virus neutralization to a greater extent than IgG. IgA antibodies measured in serum, saliva, and bronchoalveolar lavage fluid dominated in the early response to the virus. However, whilst specific neutralizing antibodies remained detectable in saliva for a long time, IgA titers in serum decreased notably a month after the onset of symptoms, keeping the long-term efficacy of this first wave response still in question. As we know, IgA is critical in the protection of mucosal surfaces against pathogens. As showed in the previous studies conducted both on influenza and parainfluenza viruses, IgA neutralizing antibodies are not only blocking the attachment of virions to the host epithelial cells but also inhibit intracellular viral replication (Mazanec et al. 1995; Sterlin et al. 2021). Likewise, the IgA response to pathogens has been widely investigated in a vast array of infections, ranging from rotavirus to human immunodeficiency virus (Blutt et al. 2012; Planque et al. 2010). Therefore, specific IgA antibodies may provide effective immunity against SARS-CoV-2 within the respiratory system, in a similar manner, that was already observed in other infectious diseases (Ejemel et al. 2020).

### Cell-Mediated Immunity

Despite the initial underestimation, there has been growing evidence indicating a critical role of T-cell adaptive immune response in the control of SARS-CoV-2. Studies on SARS-CoV-1 clearly showed the durability of cellular immunity, which was found to prevail 17 years after the infection, while antibodies titers proved to be considerably short-lived and undetectable after approximately 3–6 years post-exposure (Hellerstein 2020; Le Bert et al. 2020; Ng et al. 2016; Tang et al. 2011; Wu et al. 2007). Evidence gathered during both SARS-CoV-1 and MERS outbreaks confirm the findings regarding the novel coronavirus, suggesting that high antibody titers are associated with impaired clinical outcomes, presumably due to the extensive and uncontrollable inflammation (Hellerstein 2020; Liu et al. 2019; Lynch et al. 2021; Zhang et al. 2020), Additionally, low lymphocyte count, along with high levels of specific cytokines, were one of the first immunological discoveries in moderate and severe SARS-CoV-2 cases, suggesting a missing key element in the disease control, beyond the sole role of the humoral response (Cao 2020; Neidleman et al. 2021; Zhao et al. 2017).

Early and potent cellular response which rises seven days after exposure and peaks around 14th day, more precisely the activation of bystander CD8^+^ T cells was found to correlate with efficient viral clearance and therefore a mild or asymptomatic disease. Meanwhile, delayed bystander responses, together with systemic inflammation, were characteristic for subjects that required hospitalization (Bergamaschi et al. 2021; Moss 2022). More severe cases were also observed in delayed humoral response and poor antibody kinetics (Lucas et al. 2020a, 2021). Low CD8^+^ T cell count was found to be an independent mortality-related risk factor in SARS-CoV-2 infection while higher proportions of specific CD8^+^ lymphocytes were associated with milder cases, suggesting their role in mitigating the disease severity (Kared et al. 2021; Luo et al. 2020; Neidleman et al. 2021; Peng et al. 2020; Schulien et al. 2021; Sekine et al. 2020). Overall, an effective and sustainable neutralizing antibody protection along with broad and functional CD8^+^ T-cell response is associated with low inflammation and early recovery. What is more, CD8^+^ lymphocytes developed during COVID-19 were found to specifically differentiate into stem cells or transitional memory states, which may be crucial in forming durable protection (Kared et al. 2021). Individuals with the asymptomatic disease were found to have a highly balanced secretion of IFN-γ, IL-2, IL-10, and other inflammatory mediators, while a disproportionate secretion of pro-inflammatory cytokines was typical for symptomatic patients (Le Bert et al. 2021; Mathew et al. 2020). Numerous studies showed that CD4^+^ and CD8^+^ T cells in patients with a severe course of disease present a disrupted status of activation and function, with high concentrations of genes encoding pro-inflammatory cytokines (de Candia et al. 2021; Xu et al. 2020). Dysregulation of T helper 17 cells was found to enhance the expression of IL-17 in the lungs and thus promote the production of pro-inflammatory cytokines, which elevated levels correlated positively with the severity of COVID-19 symptoms (Sharif-Askari et al. 2022).

Interestingly, T-cell responses have been found in the majority of convalescent patients, including those with undetectable serology (Schulien et al. 2021; Sekine et al. 2020). In most human infections the presence of antibodies against a pathogen is typically regarded as a “gold standard” of immune response since antibodies are known to provide protection in the first stage of infection. However, an important concept of “cellular sensitization without seroconversion” in SARS-CoV-2 infection has emerged. Growing evidence shows that there are individuals who fail to produce virus-specific antibodies, regardless of significant exposure. However, they do develop a specific T-cell response, suggesting that a cell-mediated immune response has the potential of eliminating an infection before it fully develops (Moss 2022; Sekine et al. 2020). In addition, early studies on seronegative unexposed to SARS-CoV-2 individuals have shown that S and N-protein-specific T-cells can be found in healthy humans with no contact with the COVID-19 virus as a result of cross-reactivity with human endemic coronaviruses that cause the common cold (Le Bert et al. 2020; Pia 2020). Another theory that underpins the value of T-cell response and requires a more thorough investigation is the cell-to-cell transmission potential of SARS-CoV-2, an evasion mechanism that can explain the limitation of antibodies and complement inhibition in sera (Zeng et al. 2022).

Even though the majority of individuals develop immune response after exposure to SARS-CoV-2, whether it is humoral, cellular or both, there are cases of patients that failed to mount any immunological reaction, despite the lack of any immunodeficiencies and regardless of the severity of the disease. Therefore, providing a plausible cause for re-infections observed in some patients (Mohn et al. 2022; Nielsen et al. 2021).

While major progress has been done regarding the development of immunoassays detecting antibody responses to SARS-CoV-2, the means of assessing cell-mediated immune response are considerably less explored. However, since the IFN-γ release assay (IGRA), a well-tested and widely used tool for diagnosis of latent *Mycobacterium tuberculosis* infection, has been applied to measure IFN-γ release by antigen-specific T cells that have been developed during SARS-CoV-2 infection, the evaluation of cellular immune response has become more accessible for clinical use. As the research shows, IGRA can detect a cellular immune response to SARS-CoV-2 and therefore distinguish between convalescents and uninfected healthy blood donors with high sensitivity and accuracy. Thus, serology alone may not be sufficient in assessing the individual's protection after infection, while IGRA can serve as an invaluable diagnostic tool in the current and possibly future epidemics, especially in the assessment of patients with mild infections (Fernández-González et al. 2021; Murugesan et al. 2021; Wyllie et al. 2020).

## Vaccinations

Since the end of 2020 and the beginning of pro-vaccination campaigns all over the world, an abundance of research has emerged trying not only to assess their effectiveness but also their safety, taking into consideration the very limited timescale in which the vaccines were produced. As for today, approximately 2 years since the launch of large-scale vaccination programs, first further-reaching conclusions can be drawn. Most studies confirm the statistical reliability of the relatively high safety of currently used COVID-19 vaccines, concluding that the risk of the administration is commensurate and acceptable. The majority of adverse effects that have been observed after the first dose of the vaccine included symptoms like fever, headache and joint pain. Less frequently, cases of myocarditis or pericarditis have been reported. Among the more serious reactions that have occurred, death cases were not related to an anaphylactic or allergic reaction to the vaccine but to an aggravation of pre-existing chronic diseases (Castells et al. 2009; Fazio et al. 2022; Prakash 2022). The Pfizer/BioNTech and Moderna vaccines, both being based on the new technology of mRNA molecules, have been available on the market since the very beginning of the mass inoculation campaigns. Their efficacy in disease prevention of 95–87.5% and protection from a severe course of the infection on the level of 94.5–100%, showed the indisputable benefit of inoculation for an individual (Mascellino et al. 2021). Population wise, as far as the effectiveness of rapid, mass vaccinations to SARS-CoV-2 is concerned, the high rate of inoculation within the community was proved to be a competent tool to curb the spread of the virus (Paetzold et al. 2022). However, the decline of vaccine efficacy in preventing viral transmission over time, is one of the leading concerns regarding vaccine protection in the long term (Daković et al. 2022; Thomas et al. 2021). Moreover, the occurrence of new variants of SARS-CoV-2, such as Delta, is yet another factor that was reported to lower the vaccine-induced immunity against SARS-CoV-2 infection (Keehner et al. 2021; Nanduri et al. 2021; Rivasi et al. 2022).

According to the data available, the vast majority of individuals develop a serological response to vaccine, which was found to be stronger in the younger population and previously infected subjects. The levels of immune response to one dose of vaccination in pre-exposed subjects were comparable to two doses of vaccinations in naïve individuals (Bradley et al. 2021; Dan et al. 2021; Krammer et al. 2021; Prendecki et al. 2021; Sariol et al. 2021; Visci et al. 2022). The rapid decrease in antibody titers that were observed after SARS-CoV-2 infection, has been reported after vaccination, likewise (Visci et al. 2022). Nonetheless, it remains to be determined to what extent the antibody titers correspond with the efficacy of one’s immune response. Sariol et al. (2021) state that a decline in specific antibody titers is not commensurate to the neutralizing activity, suggesting the relevance of functional neutralizing antibody testing. Heterogeneous findings are assessing the potency of immune response elicited by vaccination in comparison with natural infection, with some reports of lower titers of antibodies after mRNA vaccine and others stating that vaccinations elicit stronger and broader immune responses than those after natural SARS-CoV-2 exposure (Altawalah 2021; Greaney et al. 2021; Richardson et al. 2022; Sariol et al. 2021). Furthermore, just as the role of cellular response to COVID-19 infection has been recently acknowledged and thoroughly investigated, so has the T-cell reaction to the vaccination. As confirmed with IGRA testing, which is slowly becoming a more popular and most importantly reliable method of quantifying T-cell response after SARS-CoV-2 infection or vaccination, cellular response is elicited in nearly all vaccinated individuals, including immunosuppressed patients, adding value to the sole serological testing (Fernández-González et al. 2021; Huzly et al. 2022; Sahin et al. 2020). Moreover, researchers state that T-cell responses are not affected by the mutations of SARS-CoV-2 and thus can provide effective protection against the new variants of the virus that escape humoral responses (Geers et al. 2021; Tarke et al. 2022).

As we approach the end of the third year of the pandemic, with possibly half of the global population infected with SARS-CoV-2 by early 2022, the promising data regarding the natural protection against the virus has emerged. Fatality rates of SARS-CoV-2 infection in 2022 have been very low, when compared to the previous years. It is yet to be determined to which extent: whether it was due to less immunogenic potential of Omicron and its subvariants that caused milder course of the disease (Pilz and Ioannidis 2022), or it was the result of a growing virus resistance after vaccination and prior infections, or both. Nonetheless, the epidemiological data confirms, that previous infections generated immunity against any SARS-CoV-2 infection that relatively waned over time (Isho et al. 2020). However, the protection against a severe course of disease remained strong. As studies show, the natural immunity may offer greater or at least equal defense against COVID-19 infection compared to one after the mRNA vaccine. The hybrid immunity, which is the combination of a previous infection and a vaccination still seems to confer the greatest protection against the disease, the benefit–cost ratio of future vaccination recommendations, especially regarding numerous vaccine boosters, should be carefully evaluated (Flacco et al. 2022; Pilz et al. 2022).

## Immune Response to Vaccine Antigens in Immunocompromised Patients

Immune response after SARS-CoV-2 vaccination among immunosuppressed patients has been of great concern to healthcare professionals in a variety of medical fields. Not only are the immunocompromised individuals a considerable number of patients, but they are also the frailest and are most likely to have a severe course of COVID-19 infection. Hence, the benefit of an effective vaccination in this cohort of patients is invaluable. Among the liver transplantation recipients that were under immunosuppressive regimens, almost half of the patients failed to develop a neither humoral nor a cellular response to the vaccines. The adjustment of drug regimens, together with the administration of calcineurin inhibitors have provided the best immune response potential, in contrast to multi‐drug immunosuppressive regimens containing mycophenolate mofetil, and possibly implementing additional vaccine boosters may lead to better post-vaccination results (D’Offizi et al. 2022; Ruether et al. 2022). Correspondingly, studies performed on a group of immunocompromised kidney transplant patients showed a weakened immune response. Nonetheless, a number of seronegative patients did develop T-cell responses, suggesting the development of partial immunity, that may limit the infection, if not prevent it (Zhang et al. 2022). As far as immunomodulatory medications are concerned, studies conducted among patients with multiple sclerosis emphasized the radical difference in developing an immune reaction to vaccination depending on drug regimens, from intact to strongly reduced immune responses. Interestingly, as far as B-cell depleting therapies are concerned, the advantage of T-cell response testing over serological assays has been proved within the scope of different studies (Bock et al. 2022; Marty et al. 2022).

## Limitations and Gaps Found by the Study

While immense progress was made in our understanding of COVID-19 infection since the beginning of the pandemic, some knowledge gaps remain to be filled. The authors revealed some visible limitations in the accuracy of the assessment of protective immunity waning and duration, whether on previous infection or vaccination. Identifying the individuals in need of extra protection against the virus is still not clear and needs further investigation. Another, and possibly the most crucial issue at the moment, is the lack of a consensus on SARS-CoV-2 vaccination programs, specifically with the constant occurrence of new variants of the virus and the benefit–cost ratio of recommending multiple vaccine boosters within the population. Possibly, more research concerning individual needs for vaccinations could be valuable at the moment.

## Closing Remarks

As presenting the full complexity of the SARS-CoV-2 mechanisms and the immune responses it elicits is nearly impossible, the authors tried to outline the most current and clinically valuable data. As far as the COVID-19 infection is concerned, the value of innate immunity components must not be overlooked, as they directly affect the outcome of the disease. However, one of the main points of focus of this study was to present the role of cellular immunity as it has proved to play a vital role in the immune defense against the novel coronavirus. New available methods of marking T-cell responses, such as applying IGRA testing, should be thoroughly explored as an effective tool in the assessment of immunity post-SARS-CoV-2 infection and vaccination. Additionally, with the development of new vaccines against SARS-CoV-2, there is a great need for elaboration on effective vaccination programs in immunocompromised patients.

## Data Availability

This manuscript has no associated data or the data will not be deposited. This is a review.
